# XPS Analysis of 2- and 3-Aminothiophenol Grafted on Silicon (111) Hydride Surfaces

**DOI:** 10.3390/molecules23102712

**Published:** 2018-10-21

**Authors:** Chieh-Hua Lee, Wan-Cian Chen, Yit Lung Khung

**Affiliations:** Department of Biological Science and Technology, China Medical University, Taichung 40402, Taiwan; u105010409@cmu.edu.tw (C.-H.L.); u105010314@cmu.edu.tw (W.-C.C.)

**Keywords:** aminothiophenol, resonance effect, surface modification, nucleophilic addition, silicon (111) hydride

## Abstract

Following on from our previous study on the resonance/inductive structures of ethynylaniline, this report examines similar effects arising from resonance structures with aromatic aminothiophenol with dual electron-donating substituents. In brief, 2- and 3-aminothiophenol were thermally grafted on silicon (111) hydride substrate at 130 °C under nonpolar aprotic mesitylene. From the examination of high resolution XPS Si2p, N1s, and S2p spectrum, it was noticed that there was a strong preference of NH_2_ over SH to form Si–N linkage on the silicon hydride surface for 2-aminothiophenol. However, for 3-aminothiophenol, there was a switch in reactivity of the silicon hydride toward SH group. This was attributed to the antagonistic and cooperative resonance effects for 2- and 3-aminothiophenol, respectively. The data strongly suggested that the net resonance of the benzylic-based compound could have played an important role in the net distribution of negative charge along the benzylic framework and subsequently influenced the outcome of the surface reaction. To the best of the authors’ knowledge, this correlation between dual electron-donating substituents and the outcome of the nucleophilic addition toward silicon hydride surfaces has not been described before in literature.

## 1. Introduction

Nucleophilic addition to silicon-hydrogenated surfaces has always been an interesting alternative to classical hydrosilylation surface reaction on silicon-hydrogenated substrates [1,2,3,4]. Although the stronger Si–C linkage via hydrosilylation have definitely attracted more attention due to the stability of the chemical linkage to silicon surfaces [5,6,7,8,9], the feasibility of direct nucleophilic addition has often been overlooked. While the chemistry of hydrosilylation has been heavily investigated over the years, reaching a general consensus amongst researchers with regard to the precise chemical mechanism has been somewhat challenging, especially for different reaction types (thermal, photoionization, or catalyst-driven). Some of these descriptions may range from simple silicon–hydride homolysis to complex exciton-based mechanism as proposed in recent years [10]. Furthermore, in many hydrosilylation reaction setups, grafting of bifunctional molecules can be rather limited in choice, especially with regard to providing useful distal functionalities. For instance, bifunctional compound with both unsaturated carbon (alkene/alkyne) and potential nucleophiles, such as OH and NH_2_, are often avoided to minimize the risk of attachment to silicon hydride surfaces via direct nucleophilic addition that can outcompete the more preferred route of reacting through the unsaturated carbon end (alkynes and alkenes) [11]. Such reaction interference has been well-recognized in early reports, such as those from Bitzer et al. [12] and Rummel et al. [13]. Hence, many groups have to tread carefully along these concepts when designing self-assembled monolayers on silicon substrates by introducing protective groups for nucleophilic moieties to avoid the “undesirable” reaction of nucleophiles directly to silicon surfaces [14,15].

In reality, such direct nucleophilic addition to silicon-hydrogenated surfaces has already been examined with interest by various groups in the past [1,2,3,16]. In fact, many of our previous reports have suggested that under elevated thermal conditions, such nucleophilic groups possess a higher degree of preference compared to the nominal formation of Si–C linkage when introduced at equimolar concentrations [4,17,18,19,20]. In view of the potential charge-trapping on the surface from nucleophiles, such interaction with nucleophilic moieties during the self-assembly of organic monolayer has often been deemed detrimental for many electronic-based applications. However, in terms of other intended applications such as biological interactions, these concerns may not be much of an issue. In view of the fact that many of these surface monolayers are intended for biological applications, silane grafting via the Si–O–Si linkages on the silicon/silica surface has frequently provided a rapid means of grafting desirable functionalities [21,22,23]. Hence, there is very little impetus to restrict the grafting of useful functional groups solely via the more stable Si–C linkage using conventional hydrosilylation methodologies. It must also be noted that the reaction conditions for hydrosilylation to obtain high-quality Si–C monolayer graft is very stringent as the process is highly susceptible to oxidation. Thus, the process of producing stable monolayers through hydrosilylation via the more stable Si–C surface linkage for many biological applications may sometimes be too tedious and challenging. Furthermore, as mentioned earlier, useful bioactive functional moieties such as amine (NH_2_) cannot be directly introduced to silicon hydride surfaces via conventional hydrosilylation methodologies due to the risk of nucleophilic additions. Hence, multiple reaction steps are often necessary to graft useful bioactive functionalities using hydrosilylation chemistry. By contrast, nucleophilic addition of short bifunctional molecules to silicon hydride surfaces is perceived to be more straightforward and simple.

While nucleophilic addition of short aliphatic chains carrying end group nucleophiles, such as NH_2_ and OH, to silicon hydride surfaces can be a quick and simple process, what remains unclear is the behavior of nucleophilic moieties when they are assigned as substituents in aromatized compounds. This is an interesting but somewhat overlooked aspect in terms of nucleophilic addition to silicon hydride surfaces. The way in which the interplay of resonance effects from aromatized compounds influences reaction outcomes had not been examined until our recent studies that looked at such effects by reacting various derivatives of ethynylaniline under elevated temperatures [20]. In our research, we observed a direct correlation between the resonance and inductive effects and subsequently identified the precise site of nucleophilic addition on the benzylic ring. Depending on how the electron-withdrawing group (EWG) and the electron-donating group (EDG) is positioned along the benzylic framework, the silicon hydride surfaces was found to have reactivity preferences in response to the resonance/inductive forces from the aromatic ethynylaniline molecule. The research also confirmed that the positioning of an EDG (NH_2_) and EWG (acetylene) along the benzylic framework would lead to either cooperation or antagonistic resonance. This cooperative or antagonistic nature would ultimately affect the preference of functional moieties’ reactivity toward silicon hydride surfaces. These observations were very much in agreement with the empirical description made by Hammetts and Hansch on resonance/inductive effects [24]. Our study on ethynylaniline was also the first time that a direct correlation of multiple substituents on aromatic benzylic system was made on grafting of silicon hydride surfaces. 

**Figure 1:**
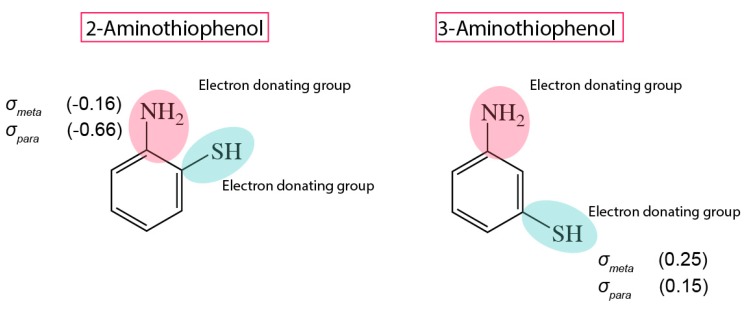
Chemical structure of 2-aminothiophenol and 3-aminothiophenol. The Hammett’s constants of the respective substituents are displayed beside the chemical groups.

In this report, to further understand and expand on the elegant interplay of resonance/inductive effect, we grafted 2- and 3-aminothiophenol on silicon (111)-hydrogenated surfaces and further examined the outcome via highly detailed XPS analysis. It is important to state that both NH_2_ and SH substituents in this study were effectively EDG, although the potential for electron-donating differed between the two species as the NH_2_ group is considered as a stronger electron-donating group compared to SH (Figure 1) [24]. During the course of this investigation, it was also observed that depending on the positioning between both groups, the aromatic system would adopt either a cooperative or antagonistic resonance effect and that this in turn helped to predict the preference of forming either a Si–N or Si–S linkage.

## 2. Results

In principle, under thermal reaction conditions that do not exceed 160 °C, the potential of silicon–hydride homolysis and the recovery of a surface radical can technically be ruled out. Therefore, direct nucleophilic addition is deemed as the only viable means for surface grafting. The addition of the lone pair from NH_2_ directly onto the silicon hydride surface will typically result in the formation of a stable Si–N type linkage. In our previous studies, we were able to examine and confirm the Si–N bonding via high resolution Si2p XPS spectra. Hence, in this report, we carefully analyzed the XPS Si2p spectrum first to attain a preliminary outlook on the reaction of 2- and 3-aminothiophenol. 

**Figure 2:**
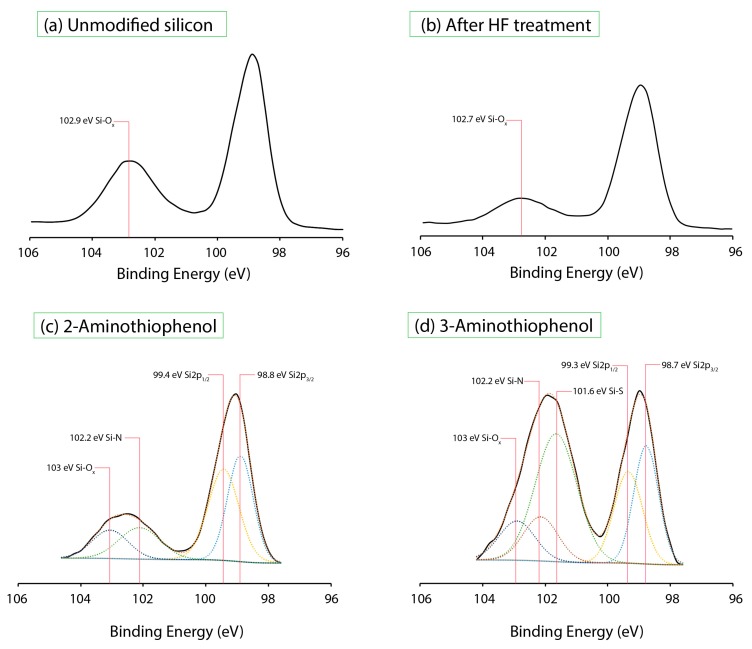
High resolution XPS Si2p spectra for (**a**) unmodified silicon (111), (**b**) after 5% hydrofluoric acid (HF) treatment for 30 s, (**c**) and (**d**) 2-aminothiophenol and 3-aminothiophenol after thermal grafting at 130 °C for 18 h on silicon (111) hydride surfaces.

The high-resolution XPS Si2p spectrums of unmodified silicon and after hydrofluoric acid (HF) treatment was deconvoluted, as shown in Figure 2a,b. The positioning of the Si–O_x_ was determined between 102.7–102.9 eV, and this helped to serve as a guide for our subsequent analysis of aminothiophenol-modified surfaces. As our XPS instrument was not found on-site, sample spectrums were not obtained immediately after the HF treatment, and this subsequently resulted in a slight increment in the oxidation level (102.7–102.9 eV), even for the Si–H surfaces (Figure 2b). For the 2-aminothiophenol-grafted silicon (111) surfaces (Figure 2c), apart from the nominal Si2p_3/2_ and Si2p_1/2_ peaks at 98.8 eV and 99.4 eV, respectively, deconvolution of the spectra was able to reveal two peaks at 102.2 eV and 103 eV. We had taken into consideration the fact that the peak centering at 103 eV was much broader and unsymmetrical compared than the Si–O_x_ peak obtained earlier from the unmodified silicon surfaces and decided that this shape must have come from a secondary peak (102.2 eV). While the assignment of the peak at 103 eV was ascribed to Si–O_x_, we attributed the peak at 102.2 eV to be that of the Si–N linkage based on previous studies [4,25]. For 3-aminothiophenol, the emergence of a large peak centering at 101.6 eV for 3-aminothiophenol was indicative of the formation of Si–S linkage on silicon substrate due to a significant shift of 0.6 eV from 102.2 eV (Si–N). It is important to note that the position of 101.6 eV is usually indicative of a conventional Si–C linkage for standard hydrosilylation for alkene/alkyne systems. However, considering the fact that both carbon and sulfur have similar electronegativity profiles, this peak may be assigned to Si–S in view of the similarity in electrostatic exertion from the silicon atom. While the observed chemical shift of the Si–N (2-aminothiophenol) from 102.2 eV to 101.6 eV for the Si–S (3-aminothiophenol) was deemed too significant, we felt that it was necessary to also accommodate deconvolution of Si–N for 3-aminothiophenol in our XPS (see Figure 2d). By comparing the area under the peak for both Si–N and Si–S, it was determined that a majority of the surface binding for 3-aminothiophenol should occur principally via the Si–S. On both surfaces, oxidation was relatively similar and considering the fact that the grafted molecule was of an aromatic nature, it was perceivable that there was sufficient steric hindrance conferred from each of the bounded molecule; this could help explain the incomplete surface coverage unlike previous reports [26].

**Figure 3:**
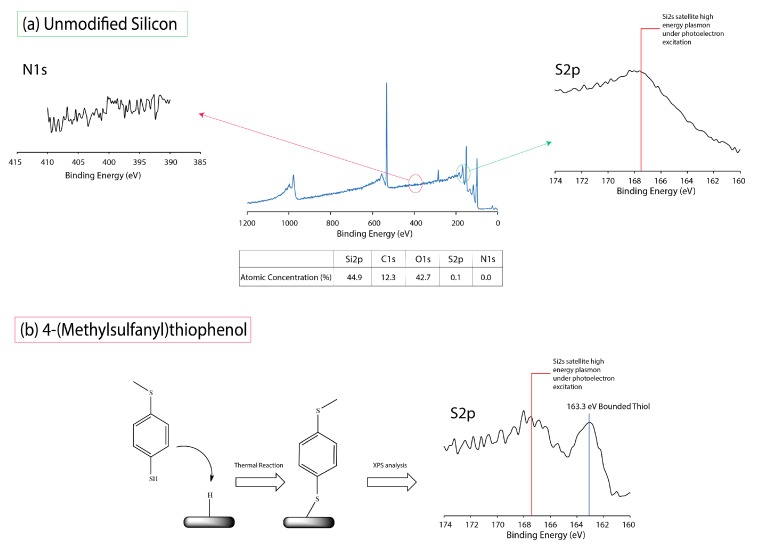
Survey spectrum, high-resolution XPS S2p and N1s spectra for (**a**) unmodified silicon (111) and (**b**) the deliberate grafting of 4-(methylsulfanyl)thiophenol for elucidating the S2p peak (163.3 eV).

An examination of the S2p spectra was deemed necessary to understand how the reaction differed between both molecules. In most situations, interpreting S2p on silicon (111) substrate is challenging due to the satellite peaks induced from the nearby Si2s (~152 eV), which is a result of surface plasmon (silicon’s plasmon energy is ~17 eV) being excited by the photoelectrons [27,28,29]. Hence, obtaining a clean S2p peak is often difficult on silicon surfaces. However, in order to better discern the S2p spectrum for Si–S binding, we decided to graft a thiol presenting 4-(methylsulfanyl)thiophenol on silicon surface. As shown in Figure 3a, on unmodified silicon surface, we observed a nominal Si2s satellite drift centering at 167.3 eV. However, upon reaction with 4-(methylsulfanyl)thiophenol, there was an emergence of a peak at 163.3 eV, and this was in turn taken as a guide for our subsequent S2p XPS analysis for 2- and 3-aminothiophenol.

**Figure 4:**
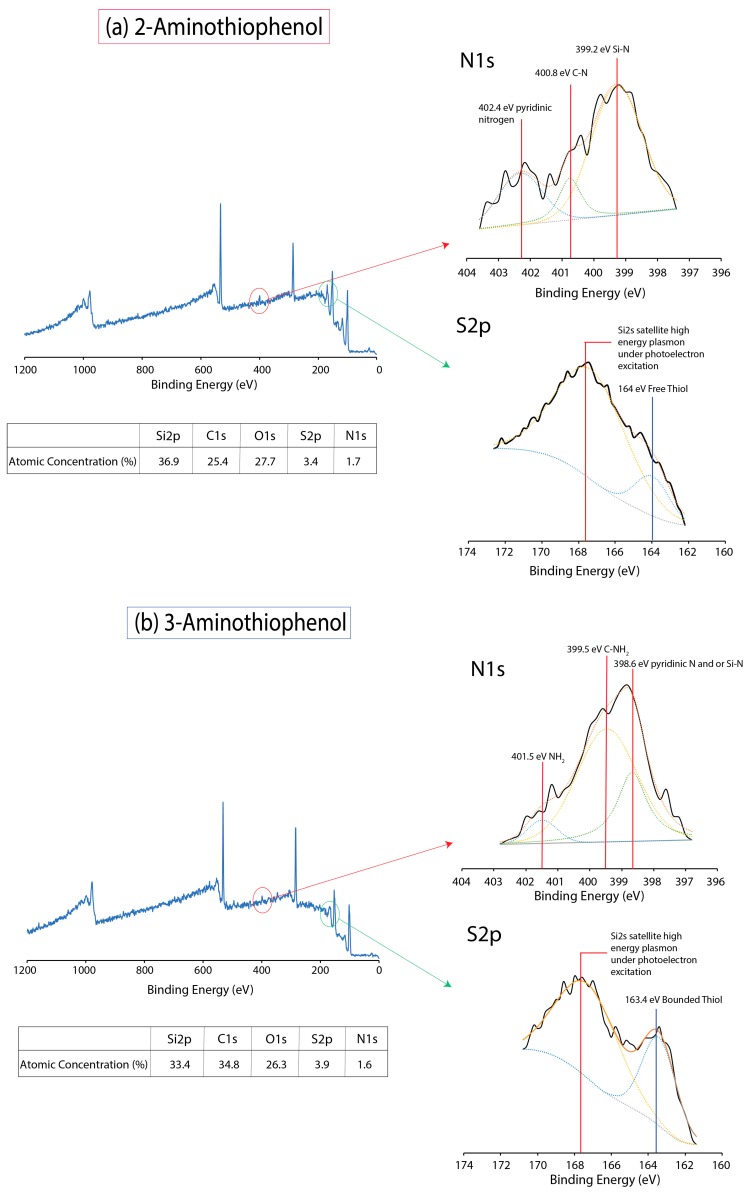
XPS survey spectrum for (**a**) 2-aminothiophenol and (**b**) 3-aminothiophenol after thermal grafting on silicon (111) hydride surfaces. Their respective N1s and S2p are shown as insets on the right.

As usual, high-resolution S2p for 2-aminothiophenol showed a Si2s-induced satellite drift similar to that of the unmodified surfaces. Hence, we were certain that the peak at 163.3 eV was one of a surface-bonded thiol; if it was attributed to merely a signature from SH group, it would certainly be present for both 4-(methylsulfanyl)thiophenol and 2-aminothiophenol. Furthermore, as shown in Figure 4b, we managed to elucidate the emergence of a peak centering at 163.4 eV once again for 3-aminothiophenol, and on the basis of our earlier analysis with 4-(methylsulfanyl)thiophenol, it was properly assigned to bounded SH on the silicon surface [30]. Furthermore, in view of the intensity of peak at 163.4 eV, it was possible to note that in the reaction of 3-aminothiophenol, a larger proportion of the molecule had reacted with the silicon hydride surface via the thiol group to form Si–S; this was consistent with our Si2p analysis (Figure 2).

As the Si2p and S2p analysis had strongly indicated that 2-aminothiophenol had grafted to the surface via Si–N linkage while 3-aminothiophenol had reacted to the surface via Si-S bond, it would be highly probable that similar observations could be drawn from the XPS N1s spectrum. In Figure 4a,b, three principle peaks can be deconvoluted for 3-aminothiophenol. The peak at 399.2 eV has previously been reported as a feature for surface-bounded nitrogen [4,31,32], while 402.2 has been assigned to pyridinic nitrogen [33,34,35], which is most likely a feature of NH_2_ moiety next to a benzylic system. On the other hand, a large peak centering at 399.5 eV and at 401.5 eV for 3-aminothiophenol (Figure 4b) was observed, and this was typically associated with free amine [36,37]. This was also an important indication that much of the NH_2_ had not actively participated in the reaction to the surface. In view of earlier Si2p analysis where we deconvoluted and assigned a smaller peak for the Si–N linkage, we carefully assigned a smaller peak at 398.6 eV accordingly for the Si–N bond. This could be either a fraction of NH_2_ in 3-aminothiophenol binding onto the silicon surface or from the mesomeric effect of the benzylic structure giving rise to pyridinic-natured nitrogen. While it would be useful to make a direct comparison to determine the degree of surface grafting between SH and NH_2_, we were largely handicapped by the huge Si2s satellite drift arising within the S2p spectrum region, and this rendered any useful and predictive measurements technically difficult. It is also necessary to state that due to the reaction conditions by which aminothiophenol compounds were diluted in mesitylene, it might not have been possible to produce a completely passivated surface, not to mention that the steric hindrance from the benzylic system would simply rule out this possibility as well. However, it was not our intention from the start to produce a fully passivated surface but rather to observe the reaction outcome of a resonating molecule with dual EDG moieties.

**Figure 5:**
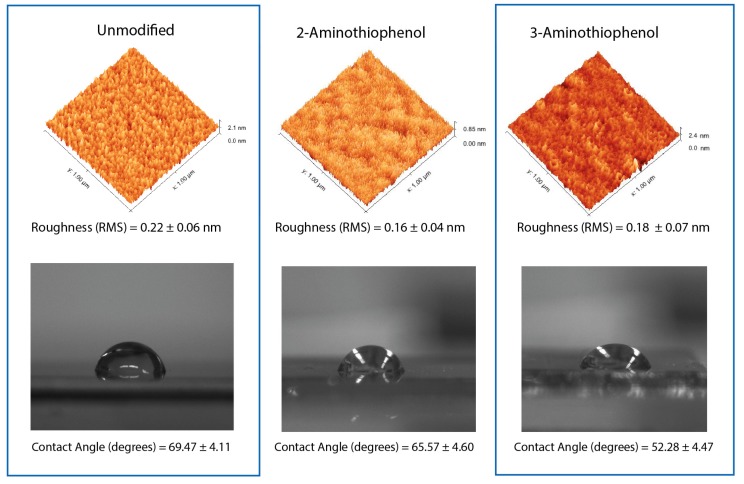
Atomic force microscopy analysis of the respective grafting with an unmodified Si (111) surface serving as a control. Roughness (rms) values and the sessile droplet angles of the surfaces are as shown below.

Atomic force microscopy (AFM) was also performed on these surface with an unmodified silicon (111) substrate as control, as shown in Figure 5. Firstly, it is important to state that our surfaces only underwent 30 s of treatment with dilute HF to remove the oxide layer; hence, we did not notice the formation of atomically flat silicon as reported previously [38]. From the roughness analysis, we did not notice any notable changes with the surface roughness compared to the silicon control surfaces. The lack of notable roughening was in agreement with our previous study on thermally grafted variants of ethynylaniline on silicon surface [20]. However, we did notice that there was a slight change in hydrophobicity profile between 2- and 3-aminothiophenol. 2-aminothiophenol registered a sessile droplet angle of 65.57 ± 4.60°, while 3-aminothiophenol surfaces had a slightly lower contact angle at 52.28 ± 4.47°. This may be attributed to the meta-positioning of NH_2_ that would be slightly raised toward the surface (3-aminothiophenol). However, more importantly, this may also have been an important indicator that both the aminothiophenols had been covalently grafted to the surface. This would then help to rule out mere physical adsorption as such events would not have given rise to any notable changes in sessile droplet measurements.

## 3. Discussion

**Figure 6:**
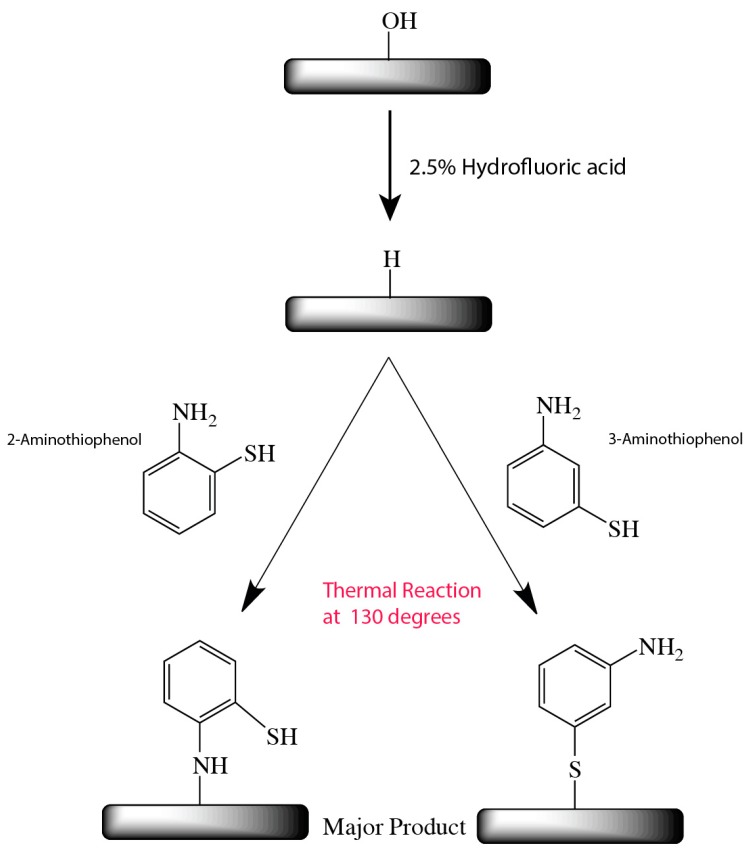
Reaction outcome for thermal grafting of 2-aminothiophenol and 3-aminothiophenol on silicon (111) hydride surface.

On the basis of the above XPS analysis, we decided to propose the following mechanism that would describe the outcome of thermally grafting 2-aminothiophenol and 3-aminothiopehnol on silicon (111) hydride surfaces. As shown in Figure 6, 2-aminothiophenol seemingly favored surface reaction via the NH_2_ group. On the other hand, 3-aminothiophenol was observed to predominantly react via the SH groups. Under low temperature (130 °C), it was thought that there would be no homolysis of the silicon hydride bond and that the nucleophilic NH_2_ and SH would react to the surface via direct nucleophilic addition mechanism. It is also important to note that hydrogen abstraction is a viable means of generating active radicals on the surface for reaction to the incoming aminothiophenol molecules [17,18,19,39,40]. However, considering that the Si2p analysis of 2-aminothiophenol did not display any appreciable Si–S linkage, this process was considered to be either relatively minimal or the nucleophilic addition reaction was the dominant process during the surface grafting.

**Figure 7:**
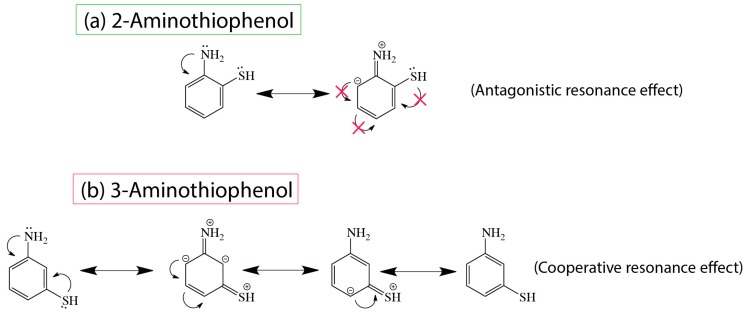
The resonance structures of (**a**) 2-aminothiophenol and (**b**) 3-aminothiophenol.

In order to provide reasons for this observed outcome, it is necessary to describe the resonance effects for the positioning of both substituents (NH_2_ and SH). When both EDG groups were positioned at ortho/meta relative to one another, it was most likely unfeasible for 2-aminothiophenol to adopt resonance due to the fact that any charge transfer along the benzylic ring would result in incompatible charge repulsion (see Figure 7). This resulted in the retention of the native lone pair on each of the substituents with little resonating effects. Because this is a nucleophilic addition reaction, the choice of solvent was highly crucial and we therefore decided on mesitylene—an aprotic solvent—so as to reduce interference with the nucleophilicity of the substituents. Polar protic/aprotic solvents are not generally considered to be useful as the solvation effects may further enhance the nucleophilicity of the larger SH, and thus very little useful information would be obtained under such conditions. Furthermore, it is also highly possible that polar solvents would interfere with the reaction on the silicon hydride surfaces [19,39,41].

In summary, when 2-aminothiophenol was thermally reacted to the surface, due to the antagonistic resonance nature, the reaction was found to be in preference toward NH_2_. On the other hand, 3-aminothiophenol experienced a cooperative resonance effect (see Figure 7b), and this might have resulted in an increase in charge concentration around the SH group that subsequently favored surface bond formation via the SH group. This was reflected by the huge Si2p peak centering at 101.6 eV as well as the emergence of a new S2p (163.4 eV) peak before the S2s plasmonic satellite feature. Furthermore, considering the difficulty in cataloguing S2p on silicon substrate, this is deemed as an important finding in this report.

## 4. Methods and Materials

*P*-type (111) boron-doped silicon (0.001–0.005 Ω cm) wafer were used, and they were purchased from Semiconductor Wafer, Inc. (SWI). Unless otherwise stated, all reagents were purchased from Sigma-Aldrich and were used as received without further purification. 

### 4.1. Thermal Reaction Protocol

In a glass reactor setup that was similar to our previous reports [4,17,18,19,20,40,42], silicon wafers were sectioned via a diamond cutter at pieces measuring approximately 10 × 10 mm^2^ in size. Prior to the reaction, the surfaces were cleaned for 30 min in hot Piranha solution (95 °C, 1 vol. 33% aqueous hydrogen peroxide to 3 vol. 95–97% sulfuric acid). The surface was then carefully removed and washed with copious amount of water before the surfaces were hydride-terminated in an aqueous solution of 5% hydrofluoric acid for a duration of 30 s. Subsequently, the samples were dipped into the respective aminothiophenol solution and (0.1 M in mesitylene) inside a custom-made Schlenk reactor vessel and the solution subsequently underwent a minimum of 15 freeze–pump–thaw cycle to remove most of the oxygen. The sample was kept under inert argon and immersed in an oil bath set to 130 °C for 18 h. The surfaces were then retrieved and were washed via sonication in copious amounts of methanol, ethanol, and chloroform before being stored in vacuum prior to XPS and atomic force microscopy analysis. The reaction of 4-(methylsulfanyl)thiophenol followed in similar fashion.

### 4.2. Atomic Force Microscopy

Atomic force microscopy (AFM) analysis of the surface were performed on a Digital Instrument NS4/D3100CL/MultiMode Scanning Probe Microscope running an in-build AFM tapping mode with cantilever tuned at frequency150 kHz, force 5 N/m, and all surfaces were analyzed in triplicates. The scan area on the surfaces was set at an area of 1 μm × 1 μm, and the scan speed was set at 0.8 Hz with the integral and proportional gain set at automatic mode. Post-image processing was subsequently performed using Gwyddion MacOS version 2.38.

### 4.3. X-ray Photoelectron Spectroscopy (XPS)

X-ray photoelectron spectroscopy analysis of the surfaces were performed on a PHI 5000 VersaProbe (ULVAC-PHI) with an Al Kα X-ray source (1486.6 eV) and taken at an angle of 45° relative to aminothiophenol-modified surfaces. Spectra were also obtained for the C1s, S2p, Si2p, and N1s in high resolution scans for all samples. The spectra were subsequently analyzed and deconvoluted using XPSpeak, while the atomic concentration was determined by CasaXPS (version 2.3.18, Casa Software Ltd., Wilmslow, UK) from binning the respective regions in the survey spectrum.

## 5. Conclusions

Aromatic molecular grafts on silicon surface can be very useful for a wide range of applications. In this report, we were able to report on the effects of resonance of dual electron-donating substituents at different positions of the benzylic ring. By appreciating the overall resonance structures, we demonstrated that we could predictably determine how an aromatic dual substituent molecule would react to silicon hydride on the basis of the resonance. This is especially useful on silicon substrate as very few reports have previously been published on the subject matter. Furthermore, this report closely corroborated with our previous reports on competitive EDG/EWG aromatic system under similar conditions, and all our current findings so far were in tandem with classical notion of dual substituent resonance.

We were able to elucidate extremely useful information from the S2p region on silicon substrate. This has not been widely reported in literature due to the overlapping of S2p region by the Si2s plasmon energies. To the best of our knowledge, the correlation between dual electron-donating substituents in a benzylic system and the direct nucleophilic addition to silicon hydride surface at elevated temperatures had not been demonstrated before. Thus, this work represents an important step toward understanding the interplay between intrinsic resonance structure and nucleophilic addition to silicon hydride surfaces and can facilitate as a platform for the selection of aromatic compounds to be grafted onto silicon hydride surfaces via nucleophilic addition.
